# An open-label, parallel-group, randomised controlled trial of antiseptic mouthwash versus antibiotics for oropharyngeal gonorrhoea treatment (OMEGA2)

**DOI:** 10.1038/s41598-020-76184-1

**Published:** 2020-11-09

**Authors:** Eric P. F. Chow, Kate Maddaford, Jane S. Hocking, Catriona S. Bradshaw, Rebecca Wigan, Marcus Y. Chen, Benjamin P. Howden, Deborah A. Williamson, Christopher K. Fairley

**Affiliations:** 1 Melbourne Sexual Health Centre, Alfred Health, Melbourne, VIC Australia; 2 Central Clinical School, Monash University, Melbourne, VIC Australia; 3 Melbourne School of Population and Global Health, The University of Melbourne, Carlton, VIC Australia; 4 Microbiological Diagnostic Unit Public Health Laboratory, Department of Microbiology and Immunology, The University of Melbourne at The Peter Doherty Institute for Infection and Immunity, Melbourne, VIC Australia; 5 Department of Microbiology, Royal Melbourne Hospital, Melbourne Health, Melbourne, VIC Australia

**Keywords:** Infectious diseases, Bacterial infection, Oral diseases, Epidemiology, Clinical trial design

## Abstract

New treatments for oropharyngeal gonorrhoea are required to address rising antimicrobial resistance. We aimed to examine the efficacy of a 14-day course of mouthwash twice daily compared to standard treatment (antibiotic) for the treatment of oropharyngeal gonorrhoea. The OMEGA2 trial was a parallel-group and open-labelled randomised controlled trial among men with untreated oropharyngeal gonorrhoea that was conducted between September 2018 and February 2020 at Melbourne Sexual Health Centre in Australia. Men were randomised to the intervention (rinsing, gargling and spraying mouthwash twice daily for 14 days) or control (standard treatment) arm and followed for 28 days. Participants in both arms were advised to abstain from sex and kissing with anyone for 14 days after enrolment. Oropharyngeal swabs were collected at baseline, Day 14 and Day 28 and tested for *Neisseria gonorrhoeae* by nucleic acid amplification test (NAAT) and culture. The primary outcome was the detection of oropharyngeal *N. gonorrhoeae* by NAAT at Day 14 after treatment. This trial was registered on the Australian and New Zealand Clinical Trials Registry (ACTRN12618001380280). This trial stopped early due to a high failure rate in the mouthwash arm. Twelve men were randomly assigned to either mouthwash (n = 6) or standard treatment (n = 6). Of the 11 men who returned at Day 14, the cure rate for oropharyngeal gonorrhoea in the mouthwash arm was 20% (95% CI 1–72%; 1/5) and in the standard treatment arm was 100% (95% CI 54–100%; 6/6). A 14-day course of mouthwash failed to cure a high proportion of oropharyngeal gonorrhoea cases.

## Introduction

An increase in antimicrobial-resistant *Neisseria gonorrhoeae* has been observed in many countries over the last decade^1–4^. In some settings, *N. gonorrhoeae* has become resistant to all available first-line therapy, and both multidrug-resistant (MDR) and extensively drug resistant (XDR) *N. gonorrhoeae* cases have been reported^5^. It is suggested that the oropharynx plays an important role in gonorrhoea transmission due to previously unrecognised practices such as kissing^6–9^. Furthermore, the oropharynx is also responsible for the emerging antimicrobial-resistant *N. gonorrhoeae* due to the poor antibiotic penetration into the oropharyngeal mucosa^10,11^.

A meta-analysis published in 2020 has concluded that the treatment efficacy of emerging treatment regimens (e.g. zoliflodacin and gepotidacin) is lower (88.8%; 95%CI 76.9–97.5%) compared with the current treatment regimens (99.1%; 95%CI 93.9–100%)^12^. Hence, it is important to develop novel interventions or alternative effective treatment for the prevention and control of gonorrhoea, particularly targeting the oropharynx^11^. In 2017, a small randomised trial and an in vitro study have suggested that antiseptic mouthwash have a significant inhibitory effect against oropharyngeal *N. gonorrhoeae* after 1 min of exposure to mouthwash^13^. This led to the OMEGA (Oral Mouthwash use to Eradicate GonorrhoeA) trial in Australia which was the first randomised controlled trial examining whether daily mouthwash use could prevent the acquisition of gonorrhoea in men who have sex with men (MSM)^14^. However, the OMEGA trial found that there was no difference in oropharyngeal gonorrhoea positivity between the intervention (Listerine) and control mouthwash (Biotène) over a 12-week period^15^. Additionally, another similar mouthwash trial for STI (sexually transmitted infection) prevention (PReGo) in Belgium has been completed in June 2020 (NCT03881007, registered at ClinicalTrials.gov). Both the OMEGA and PReGo trials examined whether mouthwash could be used for gonorrhoea prevention but did not assess the efficacy of mouthwash as an alternative treatment for oropharyngeal gonorrhoea.

Our aim was to determine the efficacy of using antiseptic mouthwash twice daily for 14 days for the treatment of oropharyngeal gonorrhoea in men and compare it to the standard antibiotic treatment.

## Methods

### Study design

The OMEGA2 (Oral Mouthwash use to Eradicate GonorrhoeA 2) trial was an open-labelled, parallel-group, randomised controlled trial comparing the treatment efficacy of using antiseptic mouthwash versus standard treatment for oropharyngeal gonorrhoea in men over a 28-day period. There were three study visits during the trial: Day 0 (baseline), Day 14 (follow-up visit 1) and Day 28 (follow-up visit 2). The OMEGA2 trial was conducted at the Melbourne Sexual Health Centre, which is the largest sexual health clinic in Victoria, Australia, between 27 September 2018, and 20 February 2020. This trial was registered on the Australian and New Zealand Clinical Trials Registry (ACTRN12618001380280) on 16 August 2018, and reported in accordance to the Consolidated Standards of Reporting Trials (CONSORT) Guidelines.

### Participants

Individuals were eligible for the OMEGA2 trial if they (a) were males; (b) were aged ≥ 16 years; (c) had been diagnosed with oropharyngeal gonorrhoea by NAAT and returned to the clinic for treatment within 7 days since their positive gonorrhoea test; and (c) had sufficient English language proficiency to understand the study procedures. Eligible criteria were not restricted by sexual orientation or HIV status. We excluded females to prevent the risk of pelvic inflammatory disease^16^. We also excluded individuals who (1) were sex workers; (2) had a positive result for other STI (e.g. chlamydia or syphilis) on the day of screening or had a positive result for gonorrhoea at other anatomical sites (i.e. urethra or anorectum); (3) had used any antibiotics within 4 weeks before the day of enrolment; (4) had a known contraindication to mouthwash; (5) had a known travelling plan; or (6) had enrolled in another clinical trial that involved other medications.

### Randomisation and treatment allocation

A computer-generated randomisation sequence with a block size of four was generated by an independent researcher. A 1:1 randomisation ratio, with no stratification, was used. Enrolled men were randomised into either the control or intervention arm. The treatment assignment was placed into an opaque sealed envelope that was labelled as per the allocation sequence and the envelopes were placed in a locked secure place.

In the control arm, men received standard treatment which was a single intramuscular injection of 500 mg ceftriaxone plus a single dose of 1 g azithromycin. On 26 August 2019, the standard treatment was changed to a single intramuscular injection of 500 mg ceftriaxone plus a single dose of 2 g azithromycin as per the Australian STI treatment guidelines^17^.

In the intervention arm, men received a bottle of alcohol-free Listerine Zero mouthwash (Johnson and Johnson, USA) labelled with their study number and a 10 mL throat spray bottle (Centaur Packaging, NSW, Australia) of alcohol-free Listerine Zero mouthwash. The brand of the mouthwash used in this trial was blinded to the participants to avoid any excessive use of mouthwash by the participants which may cause bias to the trial results. The mouthwash was repackaged into a 500 mL cleaned amber plastic bottle with a child-resistant cap and a plastic tamper-proof seal. Participants were asked to rinse and gargle 10 mL of the study mouthwash for 15 s at least twice a day, and to spray the study mouthwash 10 times to the back of their throat and then spit out any excess mouthwash at least twice a day, over 14 days. The combination of rinsing, gargling and spraying could maximize the coverage of mouthwash in the posterior oropharynx^18^. Participants were advised not to use the study mouthwash ≥ 5 times a day. We proposed a 14-day course of mouthwash use because a previous study showed that persistence of oropharyngeal gonorrhoea in MSM was 9% within 7 days after antimicrobial treatment, this dropped to 2% in 8–14 days and 1% in 15–28 days^19^. We hypothesised that a 14-day course of mouthwash might be able to reduce the load of *N. gonorrhoeae* in the oropharynx.

All participants in both arms of this trial were told to abstain from sex and kissing with anyone for 14 days after enrolment. As per the Australian STI treatment guidelines^17^, it is recommended that individuals should abstain from any sexual activity for 7 days after standard treatment. We extended this recommendation to 14 days to allow more time for the infection to be cured by the mouthwash.

### Procedures

Men with potential untreated oropharyngeal gonorrhoea were referred by clinic staff to a research nurse who performed the eligibility check. Informed consent was obtained from all enrolled participants before the commencement of the study procedures. The research nurse collected a sequentially numbered envelope and then assigned the allocated treatment to the participant.

Three oropharyngeal swabs were collected by a research nurse at each visit in the following order: (1) at the tonsillar fossae; (2) at the posterior oropharynx; and (3) at both the tonsillar fossae and posterior oropharynx. The first two swabs were tested for *N*. *gonorrhoeae* by transcription-mediated amplification (TMA) based NAAT (Aptima Combo 2, Hologic Inc., USA). The third swab was placed onto a GC Agar medium for culture. Whole genome sequencing (WGS) and bioinformatic analysis on all cultured *N. gonorrhoeae* isolates were performed (Appendix).

There was a protocol deviation on compensation during the trial. In the original protocol, participants did not receive any compensation for their participation. During routine trial monitoring, we observed slow recruitment rate and high declining rate; and thus, an AUD$100 (USD$72) gift voucher was given to participants recruited from 1 October 2019 onwards as compensation for their time and travel costs at Day 28.

### Outcomes

The primary outcome was the cure rate for oropharyngeal gonorrhoea. We defined treatment failure when a positive test for oropharyngeal gonorrhoea by NAAT or culture on Day 14. The standard treatment was then given to participants who experienced treatment failure in the mouthwash arm at Day 14. Men with an equivocal NAAT result were considered as ‘cured’ if the culture test result was negative or as a ‘treatment failure’ if the culture test result was positive at Day 14. The secondary outcomes included sexual abstinence during the 14-day treatment.

### Statistical analyses

This trial aimed to determine the proportion of men who experienced cure for oropharyngeal gonorrhoea using mouthwash twice a day for 14 days compared with standard treatment. We determined that a sample size of 100 in each arm would provide reasonable 95% confidence intervals (CI) if we hypothesised that the cure rate for oropharyngeal gonorrhoea in the mouthwash arm was 100% (95% CI 96–100%). There was a protocol deviation on performing an interim analysis when 12 participants were recruited due to the difficulties in recruitment including high declining rate and the COVID-19 pandemic. We employed an early stopping rule if the upper bound of the cure rate was < 80%, this early stopping rule has been used in other trials^20^. After the interim analysis, the trial was stopped early due to a high treatment failure rate in the mouthwash arm.

The cure rate was defined as the number of men tested negative for oropharyngeal gonorrhoea divided by the number of men tested for oropharyngeal gonorrhoea by NAAT at Day 14. The 95% CI of the cure rate was calculated using the binomial exact method. We compared the cure rate between the two arms by calculating the risk difference and the Fisher’s exact test. All statistical analyses were performed using Stata (version 14, College Station, TX: StataCorp LP).

### Ethical consideration

This trial was approved by the Alfred Hospital Ethics Committee (153/18). All procedures were performed in accordance with the National Statement on Ethical Conduct in Human Research and the Good Clinical Practice Guidelines. Given we were using a non-recommended and non-antibiotics treatment, we considered it appropriate to only recruit individuals with oropharyngeal gonorrhoea without infections at other anatomical sites to minimise the risk to participants. Oropharyngeal gonorrhoea is mostly asymptomatic, has a relatively short duration of less than 3 months and it is likely that most infections in the population go undiagnosed and clear naturally. The other concern was the potential for transmission of gonorrhoea from oropharyngeal infection that may not be adequately treated if mouthwash were less effective than antibiotics. Therefore, we recommended individuals not to kiss and have sex for 14 days instead of the standard recommendation of 7 days.

## Results

**Figure 1:**
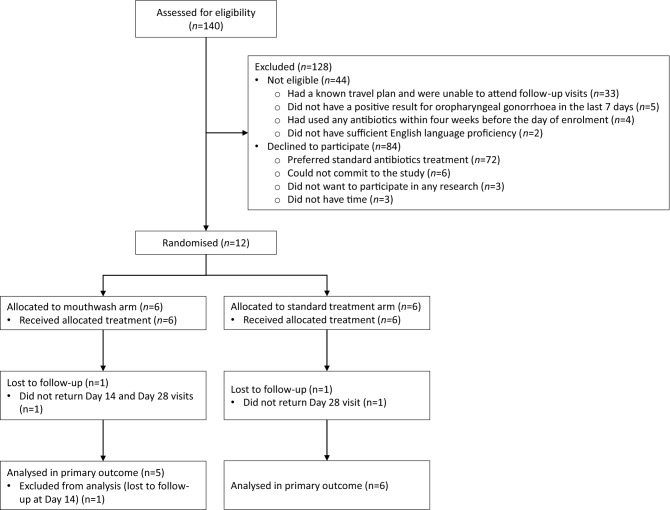
Flow diagram of study recruitment and randomisation to intervention (mouthwash) or control (standard treatment) arm.

Between September 2018 and February 2020, 140 men were referred to the research team, 44 men did not meet the inclusion criteria, 84 men were excluded, and the remaining 12 men were enrolled and randomised (Fig. 1). There was no significant difference in age between men who participated and those who were excluded or ineligible (Mann–Whitney *U* test, *p* = 0.247). The median age of the 12 participants was 32 (interquartile range (IQR) 29-37). All participants were men who have sex with men and HIV negative. The demographic characteristics were similar between the two arms (Table 1).

**Table 1:** Baseline demographic characteristics of 12 enrolled men.

	All (n = 12)	Mouthwash arm (n = 6)	Standard treatment arm (n = 6)
Age (years), mean ± standard deviation	33.2 ± 7.9	34.2 ± 10.6	32.2 ± 4.7
Currently taking HIV pre-exposure prophylaxis, *n* (%)	5 (42%)	2 (33%)	3 (50%)
Had sex with male(s) in the past 3 months, *n*(%)	12 (100%)	6 (100%)	6 (100%)
Had sex with female(s) in the past 3 months, *n*(%)	0 (0%)	0 (0%)	0 (0%)
Had ever used mouthwash, *n* (%)	10 (83%)	5 (83%)	5 (83%)

Of the 12 enrolled men, 11 (92%) men attended the follow-up visit at Day 14 and were included in the analysis (5 in the mouthwash arm and 6 in the standard treatment arm). The cure rate for oropharyngeal gonorrhoea in the mouthwash arm was 20% (95% CI 1–72%; 1/5) and in the standard treatment arm was 100% (95% CI 54–100%; 6/6), with a risk difference of − 0.80 (95% CI − 1.15 to − 0.45; *p* = 0.015). Of the four men with treatment failure in the mouthwash group, three men had a positive result for *N. gonorrhoeae* culture at baseline and Day 14 (Table 2). Bioinformatic analysis indicated these isolates likely represented the same infection (Appendix). The man who was cured in the mouthwash arm had equivocal results for NAAT at both the tonsillar fossae and posterior oropharynx at Day 14 and Day 28 but his culture test result was negative at both follow-up visits. Of the four men with treatment failure in the mouthwash group, three men received standard treatment between Day 14 and Day 28 after the positive NAAT and culture results at Day 14, and all three men tested negative for NAAT and culture at Day 28 after receiving standard treatment (Table 2). One man did not return to the clinic for standard treatment until Day 28 and his tests result for NAAT and culture remained positive for Day 14 and Day 28 (Table 2). All five men in the mouthwash arm reported using the mouthwash twice a day over the 14-days period with the recommended dose (10 mL), duration (15 s) and methods (rinse, gargle and spray) each time. No men reported an adverse event during the trial.

**Table 2:** Clinical outcome of oropharyngeal gonorrhoea of 12 enrolled men.

Study ID	Assigned treatment	Treatment outcome	Laboratory results								
			Day 0 (Baseline)			Day 14 (1st follow-up visit)			Day 28 (2nd follow-up visit)		
			Posterior oropharynx NAAT	Tonsillar fossae NAAT	Oropharyngeal culture	Posterior oropharynx NAAT	Tonsillar fossae NAAT	Oropharyngeal culture	Posterior oropharynx NAAT	Tonsillar fossae NAAT	Oropharyngeal culture
A	Standard treatment	Cure	+	+	−	−	−	−	N/A	N/A	N/A
B	Standard treatment	Cure	+	+	+	−	−	−	−	−	−
C	Standard treatment	Cure	+	+	+	−	−	−	−	−	−
D	Standard treatment	Cure	+	+	+	−	−	−	−	−	−
E	Standard treatment	Cure	−	−	−	−	−	−	−	−	−
F	Standard treatment	Cure	+	+	+	−	−	−	−	−	−
G	Mouthwash	Failure	+	+	−	+	+	+	−	−	−
H	Mouthwash	Cure	+	?	−	?	?	−	?	?	−
I	Mouthwash	Failure	+	+	+	+	+	+	+	+	+
J	Mouthwash	Unevaluable	+	+	+	N/A	N/A	N/A	N/A	N/A	N/A
K	Mouthwash	Failure	+	+	+	+	+	+	−	−	−
L	Mouthwash	Failure	+	+	+	+	+	+	?	−	−

+ Positive; − Negative; ? Equivocal; N/A Test not done.

The Study ID has been recorded to remain anonymity and confidentiality. Participant I did not attend until Day 28 for treatment, whereas Participants G, K and L were treated prior to their Day 28 visit. Participants F and L received an AUD$100 (USD$72) gift voucher as compensation for their time and travel costs at Day 28 after the change of the protocol. In the standard treatment arm, Participants A, B, C and D received 500 mg ceftriaxone plus a single dose of 1 g azithromycin; while Participant E received 500 mg ceftriaxone plus a single dose of 2 g azithromycin due to the change of the treatment guidelines.

Two out of 11 men (18%; 95% CI 23–52%) self-reported sexual contacts within 14 days after enrolment and both men were in the standard treatment arm.

## Discussion

To our knowledge, this is the first trial examining the effectiveness of antiseptic mouthwash for the treatment of oropharyngeal gonorrhoea. We found that a 14-day course of antiseptic mouthwash twice a day with rinsing, gargling and spraying was not an effective treatment with 80% of cases failing treatment. Indeed, the 95% confidence for mouthwash treatment failure was between 28 and 99%; these intervals are well above the accepted failure rate of only 5%. Although a previous mouthwash trial (the GONE [GONorrhoea Eradication] trial) was conducted on patients with confirmed oropharyngeal gonorrhoea infection, it was not an exclusive mouthwash treatment trial as only a single dose of mouthwash was used for one minute and standard treatment was provided after the mouthwash use^21^. The GONE trial found that mouthwash inhibited the growth of *N. gonorrhoeae* when samples were taken five minutes after its use^21^. Taking the results of these two trials together, it is possible that mouthwash may have a short term effect of inhibiting the growth of *N. gonorrhoeae*, but this short term effect does not translate into an effective treatment even when mouthwash is used twice a day for 14 days.

The results of the GONE trial and the current OMEGA2 trial are not necessarily contradictory. The GONE trial simply measured the proportion of men with culture positive of oropharyngeal swabs 5 min after mouthwash use^21^, and the OMEGA2 trial assessed whether mouthwash would eradicate oropharyngeal gonorrhoea over a 2-week period when it is used twice daily. The antibacterial effect of mouthwash is likely to only persist while it remains on the surface of the oropharyngeal epithelium and this period is likely to be short-lived given the fluids and substances passing over it from swallowing including saliva, food and beverages, all of which are likely to rapidly reduce its concentration and likely effect. Therefore, it is reasonable to postulate that mouthwash could have an immediate but not prolonged effect which is consistent with the results of both the GONE trial and OMEGA trial^15,21^.

There are several other factors that may have contributed to the failure of twice daily mouthwash use to effectively treat oropharyngeal gonorrhoea. First, mouthwash may not be able to reach the epithelium of the tonsillar fossae and posterior pharyngeal wall where the *N. gonorrhoeae* organisms most commonly reside. Past studies have demonstrated that poor antibiotic penetration into oropharyngeal mucosa and this may also be the case for mouthwash^22^. Our previous work using a mouthwash with food dye has shown that reaching the posterior oropharyngeal wall is difficult even with gargling and this is the reason we also asked men to spray with mouthwash^18^; however, even with 10 sprays of mouthwash, only about one-third of the posterior pharyngeal wall was visibly covered with the food dye and therefore areas where *N. gonorrhoeae* commonly occur, may have not been exposed to mouthwash with each application^18^. The inability of mouthwash to reach all surfaces of the oropharynx is also supported by the results of the GONE trial that found that there was a moderate difference in the effect of mouthwash by the site of gonorrhoea positivity at the tonsillar fossae (from 90 to 57%) versus the posterior pharynx (from 70 to 57%)^21^.

Further, it is also likely that even if the mouthwash reached the epithelium where the organisms were, it would not have reached organisms in the tonsillar crypts or those that were within cells^23^. A study collected tonsillectomy specimens from clients with oropharyngeal gonorrhoea and reviewed these histologically to determine the site of the diplococci^23^. While most bacteria were in the uppermost layers of the epithelium, some bacteria were in pouches and tonsillar crypts. Most bacteria were intracellular.

The OMEGA2 trial used an alcohol-free Listerine Zero mouthwash while the GONE trial used Listerine Cool Mint mouthwash that contained 20% alcohol^21^. Both mouthwashes were highly effective in laboratory studies that exposed a strain of *N. gonorrhoeae* to dilutions of 10^8^ colony forming units although Listerine Zero was slightly more effective and had the advantage of not containing alcohol^21^. Given that Listerine Zero failed so dramatically, it seems unlikely that other antiseptic mouthwashes would be effective for the treatment of oropharyngeal gonorrhoea.

There are several limitations to this trial. First, blinding was not possible in our trial and therefore some participants may have changed their sexual practices more in one group than another. The magnitude of the effect size between the two groups makes it unlikely that this could have materially changed the results. Second, there was a high proportion of potentially eligible men who declined to participate (66%; 84/128) but there was no significant difference in median age between men who participated and those who did not. Most men expressed they preferred the antibiotics treatment rather than the mouthwash use and they did not want to abstain from sex and kissing for 14 days, suggesting sexual abstinence for 14 days is not acceptable. This is consistent with an Australian study showing 10% of MSM resumed sex within a week after treating for anorectal chlamydia although these men were advised to abstain from sex for 7 days after treatment^24^. Third, overestimation of the treatment effect might have occurred due to early stopping of this trial^25,26^. We decided to stop the trial after 12 men were recruited not only because the high treatment failure rate in the mouthwash and high proportion of men declining to participate suggesting this treatment might not be flexible or acceptable.

To date, there has been no trial purposing and examining whether mouthwash could be used as an alternative treatment for oropharyngeal gonorrhoea. This trial, however, shows that a 14-day course of Listerine mouthwash fails to cure oropharyngeal gonorrhoea. Taking into account with the findings from existing mouthwash studies on oropharyngeal STI, it is unlikely that mouthwash can be used as an alternative intervention to prevent the acquisition or to treat *N. gonorrhoeae* in the oropharynx. The results of the GONE trial however suggest that mouthwash may reduce culture positive detection shortly after use and the utility of this observation for the control of gonorrhoea requires further work. There is a real prospect that without new treatments for gonorrhoea that it may become resistant to all known antibiotics. New antibiotics, vaccine and novel interventions are urgently needed for gonorrhoea prevention and control in the era of emerging antimicrobial resistance.

## Supplementary information

Supplementary Information 1.
