# Supplementary material for: An open-label, parallel-group, randomised controlled trial of antiseptic mouthwash versus antibiotics for oropharyngeal gonorrhoea treatment (OMEGA2)
Source: Sci Rep. 2020 Nov 9;10:19386. doi: 10.1038/s41598-020-76184-1 (PMC7652834; doi:10.1038/s41598-020-76184-1)
Supplement: Supplementary file 1 — Supplementary Information 1. [file 41598_2020_76184_MOESM1_ESM.pdf]

An open-label, parallel-group, randomised controlled trial of antiseptic mouthwash compared with antibiotics for oropharyngeal gonorrhoea treatment (OMEGA2)

Eric P.F. Chow[1,2,3], Kate Maddaford[1], Jane S Hocking[3], Catriona S Bradshaw[1,2], Rebecca Wigan,[1] Marcus Y Chen[1,2], Benjamin P. Howden[4], Deborah A Williamson[4,5], Christopher K Fairley[1,2]

1. Melbourne Sexual Health Centre, Alfred Health, Melbourne, Victoria, Australia
2. Central Clinical School, Monash University, Melbourne, Victoria Australia
3. Melbourne School of Population and Global Health, The University of Melbourne, Carlton, Victoria, Australia
4. Microbiological Diagnostic Unit Public Health Laboratory, Department of Microbiology and Immunology, The University of Melbourne at The Peter Doherty Institute for Infection and Immunity, Melbourne, Victoria, Australia
5. Department of Microbiology, Royal Melbourne Hospital, Melbourne Health, Melbourne, Victoria, Australia

SUPPLEMENTARY APPENDIX

Whole genome sequencing and bioinformatic analysis

DNA extraction, whole genome sequencing (WGS) and bioinformatic analysis of study isolates was performed at the Microbiological Diagnostic Unit Public Health Laboratory, University of Melbourne. Genomic DNA was extracted from a single colony using a QIAsymphony™ DSP DNA Mini Kit (Qiagen) according to manufacturer's instructions, and WGS was performed on an Illumina NextSeq 500 instrument with 150 bp paired-end reads using Illumina libraries and protocols (Illumina, San Diego, California, USA). Reads were trimmed to remove adaptor sequences and low-quality bases (Q<10) with Trimmomatic v0.38.[1] Kraken (v2.0.7) was used to investigate for contamination.[2] *De novo* assembly was performed using Shovill (https://github.com/tseemann/shovill), and genes were annotated using Prokka (v1.13).[3] NG-MAST (*N. gonorrhoeae* multi-antigen sequence type) STs were assigned using NGMaster v0.5.5,[4] with data from the NG-MAST database (http://www.ng-mast.net). MLST STs were identified using MLST v2.15 (https://github.com/tseemann/mlst) with the PubMLST database (http://pubmlst.org/neisseria). Isolates were also typed in silico using the *N. gonorrhoeae* sequence typing for antimicrobial resistance (NG-STAR) scheme implemented in pyngSTar (https://github.com/leosanbu/pyngSTar).[5] All sequencing data for isolates in this study are available from the NCBI Sequence Read Archive (BioProject PRJNA317462).

Overall, four individuals had paired baseline and day 14 isolates. All eight isolates underwent WGS. Of the four individuals, three had isolates at baseline and day 14 that were the same MLST, NG-MAST and NG-STAR types, and were therefore thought to represent the same infection (Supplementary Table 1).

Supplementary Table 1. Typing results from paired baseline and day 14 isolates.

Study ID	MLST		NG-MAST		NG-STAR	
	**Baseline**	**Day 14**	**Baseline**	**Day 14**	**Baseline**	**Day 14**
1	8156	8156	5441	5441	442	442
3	1599	1599	11461	11461	520	520
6	8156	8156	5441	5441	442	442

Abbreviations: MLST, multilocus sequence type; NG-MAST, *N. gonorrhoeae* multi-antigen sequence type; NG-STAR, *N. gonorrhoeae* sequence typing for antimicrobial resistance

REFERENCES

1 Bolger, A. M., Lohse, M. & Usadel, B. Trimmomatic: a flexible trimmer for Illumina sequence data. *Bioinformatics (Oxford, England)* **30**, 2114-2120, doi:10.1093/bioinformatics/btu170 (2014).

2 Wood, D. E. & Salzberg, S. L. Kraken: ultrafast metagenomic sequence classification using exact alignments. *Genome biology* **15**, R46, doi:10.1186/gb-2014-15-3-r46 (2014).

3 Seemann, T. Prokka: rapid prokaryotic genome annotation. *Bioinformatics (Oxford, England)* **30**, 2068-2069, doi:10.1093/bioinformatics/btu153 (2014).

4 Kwong JC, G. d. S. A., Dyet K, Williamson DA, Stinear TP, Howden BP, Seemann T NGMASTER: in silico multi-antigen sequence typing for Neisseria gonorrhoeae. *Microbial Genomics*, doi:10.1099/mgen.0.000076 (2016).

5 Demczuk, W. *et al.* Neisseria gonorrhoeae Sequence Typing for Antimicrobial Resistance, a Novel Antimicrobial Resistance Multilocus Typing Scheme for Tracking Global Dissemination of N. gonorrhoeae Strains. *Journal of clinical microbiology* **55**, 1454-1468, doi:10.1128/jcm.00100-17 (2017).
